# Patterns and timing of recovery from facial nerve palsy after nerve-sparing parotid surgery: the role of neuromuscular retraining

**DOI:** 10.1007/s00405-024-08758-y

**Published:** 2024-06-24

**Authors:** Giulia Molinari, Federico Calvaruso, Alice Barbazza, Elena Vanelli, Federica Nizzoli, Elena Reggiani, Monica Guidotti, Aurora Borghi, Daniele Marchioni, Livio Presutti, Ignacio Javier Fernandez

**Affiliations:** 1 Department of Otolaryngology - Head and Neck Surgery, IRCCS Azienda Ospedaliero - Universitaria di Bologna, Bologna, Italy; 2 Department of Medical and Surgical Sciences (DIMEC), Alma Mater Studiorum - University di Bologna, Bologna, Italy; 3 Department of Otorhinolaryngology - Head and Neck Surgery, University Hospital of Modena, Modena, Italy

**Keywords:** Facial nerve palsy, Facial nerve palsy rehabilitation, Neuro-Muscolar-Retraining, Parotid surgery, Major salivary glands surgery

## Abstract

**Objectives:**

Among the complications of parotid surgery, facial palsy is frequent and burdened by high functional and social impact for the patient. There are few data on the efficacy of facial neuromuscular retraining (FNR) in patients with facial palsy after parotid surgery, and no data exist on its impact in timing and extent of recovery.

**Material and methods:**

A retrospective study was conducted on patients undergoing FN sparing parotid surgery and suffering from postoperative facial palsy. Among 400 patients undergoing surgery between July 2016 and May 2023, those with the preservation of the FN and onset of facial palsy were selected. Nerve function was evaluated during 2 years follow up using the House-Brackman (H&Bs) and Sunnybrook scales (SBs).

**Results:**

A total of 46 patients undergoing partial or total parotidectomy were included. At discharge 18 patients (39,1%) had IV to VI grade paralysis according to the H&Bs and the mean SBs value was 54. At 2 and 6 months after surgery, the average value of Sunnybrook increased to 76.5 and 95.4 respectively. After 12 months no patients with IV to VI grade paralysis were represent in our cohort. Two years after surgery, only five patients (10.9%) had persistent grade II paralysis according to HBs.

**Conclusions:**

Our study supports the efficacy of FNR in the rehabilitation of facial paralysis after nerve-sparing parotidectomy. The greater functional improvement is achieved within the first 6 months of rehabilitation. A significant improvement is detected still after 18 months, supporting the importance of long rehabilitation for patients without complete recovery after the first year.

## Introduction

Transitory facial nerve (FN) palsy is among the most relevant complications of parotid surgery, with an incidence varying widely between 10 and 65% of cases [1, 2]. Dissection of the salivary parenchyma from the facial nerve branches could cause nerve functional impairment, despite the fact that the continuity of the fibers is preserved. An apparent intact FN sheath could hide non-functioning or interrupted axons (neurapraxia or axonotmesis) or even neurotmesis, caused by nerve traction or manipulation during surgery, that increase the nerve stimulation threshold and clinically determine facial nerve palsy (FNP) [3]. In such cases, since axonotmesis is potentially reversible, the chance of spontaneous complete recovery is high. However, in cases with neurotmesis the healing process could last several months and occasionally the FN function could not return to normal. A number of therapeutic approaches have been applied to post-surgical FNP, ranging from medical therapy to FN motor rehabilitation, including Neuromuscolar Retraining (NMR), a rehabilitation strategy based on active small, slow, symmetric movements and passive facial external and intraoral massages [4]. While several authors have explored risk factors for iatrogenic FNP [1, 2, 5] and on the surgical rehabilitation techniques in case of intraoperative nerve section, very few have focused on the impact of post-operative treatments on the recovery of FN palsy with an intact FN. In particular, the role of NMR after nerve-sparing parotid surgery has never been investigated.

The aim of this study was to assess the pattern and timing of recovery from FNP in a cohort of patients undergoing parotid surgery with FN anatomic preservation and rehabilitated with NMR. The impact of different clinical variables on facial nerve recovery was investigated to identify prognostic factors for complete recovery of nerve function.

## Patients and methods

### Patients

This is a retrospective review of patients treated with partial or total parotidectomy between July 2016 and May 2023 at the Department of Otorhinolaryngology-Head and Neck Surgery of the University Hospital of Modena and Bologna, two tertiary referral centres. Parotid surgery was performed in all cases by standard approach through Blair’s modified incision, under continuous operative facial monitoring with Nerve Integrity Monitor (NIM) system (Medtronic, USA). Surgery of the parotid gland was classified according to the European Salivary Gland Society (ESGS) classification system [6]. Patients developing FNP despite intraoperative preservation of facial nerve integrity, and who were sent to rehabilitation with NMR were included. Patients with normal facial function after surgery, those who underwent intraoperative section of one or more branches, or refused FN rehabilitation, were excluded.

### Assessment of post-operative facial nerve function

All patients were assessed at our institutional FNP rehabilitation clinic by a multidisciplinary team made up of an otolaryngologist and a speech therapist dedicated to FP, within a mean time of 15 days from surgery (range 2–37). The severity of the facial deficit was classified according to the House-Brackman rating scale (HBs) and the Sunnybrook scale (SBs). The first evaluates the capacity of the movements of one side of the face, and of the different branches of the facial nerve, according to six grades, from I = normal facial function to VI = complete FNP [4, 7, 8] The SBs is more complex but more specific as it comprises a global evaluation created by the study of the individual areas of the face, compared to the unaffected side [9]. It evaluates the resting symmetry, the symmetry in performing voluntary movements, and the presence or absence of synkinesis. The SBs ranges from 0 to 100, with 100 representing a completely normal facial function [4, 7, 10].

### Facial palsy rehabilitation program

The rehabilitation method through NMR was applied in the selected cohort of patients, regardless the grade of postoperative facial palsy. The patient was educated to perform selective motor control strategies, first in the presence of the dedicated speech therapist and then independently at home. The main objectives of this program are the reduction of synkinesis and facial muscles hypertonic contraction, and the improvement of facial symmetry. Moreover, the use of mirror exercises allows the sensory input to enhance the central control of the movements of single mimic muscle, and thus promote neural adaptation. The facial massages are effective in preventing post-paretic syndrome and the small movements allow reduction of recruitment and hyperactivity of adjacent muscles, with an improvement in fine muscular coordination. The slow pattern of contraction allows the patient to have adequate feedback, by carefully observing the movement and correcting the velocity or the strength of contraction, as required. Finally, the symmetry of movements allows a physiologic activation of the affected side and avoids the muscular over-contraction on the healthy side [10]. For the purpose of the present study, grade II or III FNP according to HBs was defined as “mild”, while FNP equal or worse to grade IV according to HBs was considered “severe”. After the initial treatment meeting, during which the patient was taught the NMR methodology, the patient had to practice the exercises every day at home. A complete clinical assessment of the nerve function was performed after surgery, at the beginning of rehabilitation (T0) at 2,6,12, 18 and 24 months after surgery (namely T2, T6, T12, T18 and T24). At each follow-up visit, HBs and SBs were applied, and data prospectively recorded on a digital database.

### Statistical analysis

The statistical analysis was conducted with SPSS 19.0 for Windows (IBM inc, USA) and JASP for windows version 0.16.3.0. The normal distribution of continuous variables was assessed by means of the Shapiro - Wilk test, and distribution parameters (mean, median, standard deviation, and range) were calculated. Comparison between categorical variables was carried out with the Chi square test or Fisher’s exact test as appropriate. Comparison between continuous variables with normal distribution was made with Student’s T-Test, while Mann – Whitney test was used for those without normal distribution. The temporal evolution of facial palsy evaluated with the SBs or the HBs were assessed with the repeated measure ANOVA. Variances were tested with Levene’s test for equality of variances. The multivariate analysis was conducted with linear regression analysis, considering the global value of the SBs or HBs at last available follow-up. Linear regression for Sunnybrook scale at intermediate timepoints (e.g. T2) were conducted to check for differences among variables after observing different trends in repeated ANOVA curves. Differences were considered statistically significant for *p* values *≤* 0.05 with confidence interval set at 95%.

### Ethical committe

This retrospective multicentric study was approved by the Internal Review Boards (IRB) of the University Hospitals of Modena and Bologna (901/2021/OSS/AOUMO; 160/2022/OSS/AOUBO). The study was performed according to the Declaration of Helsinki.

## Results

Among the 400 patients who underwent parotid surgery in the considered period at the two Institutions, 46 patients (16 men and 30 women) met the inclusion criteria and were followed for a mean period of 12 months (range 2–24 months). Mean age at surgery was 54 (range 17–86). Table 1 reports the details of the surgery performed. FN was reported as responding at intraoperative final stimulation in 44 pts, while in 2 cases was not responding. Unfortunately, the intensity of the final stimulation in mV was not retrievable in all patients.

**Table 1:** Surgical data regarding the operated patients. ESGS: The European Salivary Gland Society

***Operated side***	Number of patients (%)
Right	26 (56,5)
Left	20 (43,5)
***Type of parotidectomy (ESGS classification)***	
Extracapsular dissection of level I or II	4 (8,7)
Partial parotidectomy (level I and II)	11 (23,9)
Subtotal parotidectomy (level I-II-III or level I-II-IV)	4 (8,7)
Total parotidectomy (I-II-III-IV)	17 (37)
Revision surgery (totalization)	10 (21,7)
***Associated extra-parotid surgery***	
Ipsilateral neck dissection	14 (30,4)
External carotid artery	5 (10,9)
Great auricular nerve	4 (8,7)
Sternocleidomastoid muscle	1 (2,2)
Masseter muscle	1 (2,2)
Skin	1 (2,2)

**Fig. 1:**
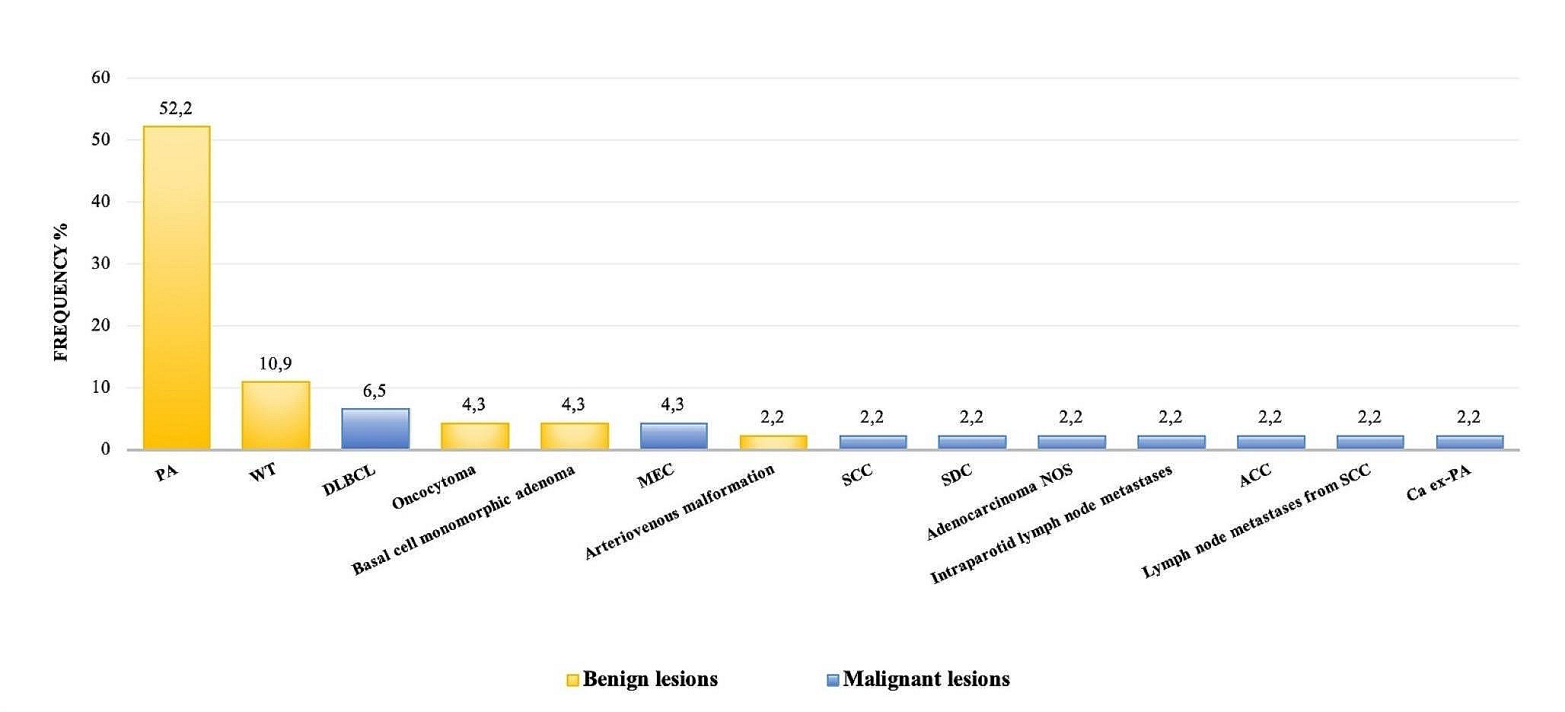
Distribution of surgically treated lesions in the cohort. PA: Pleomorphic adenoma; WT: Warthin tumour; DLBCL: Diffuse large B-cell non-Hodgkin lymphoma; MEC: Mucoepidermoid carcinoma; SCC: Squamous cell carcinoma; SDC: Salivary duct carcinoma; ACC: Acinic cell carcinoma; Ca ex-PA: Carcinoma ex pleomorphic adenoma

The histological diagnosis of the surgically removed lesions mostly consisted of neoplastic lesions in 45 patients (98%), of which 12 were malignant and 33 benign, as detailed in Fig. 1. It is important to specify that the patients included in the study who had malignancies did not undergo sacrifice of the facial nerve.

**Fig. 2:**
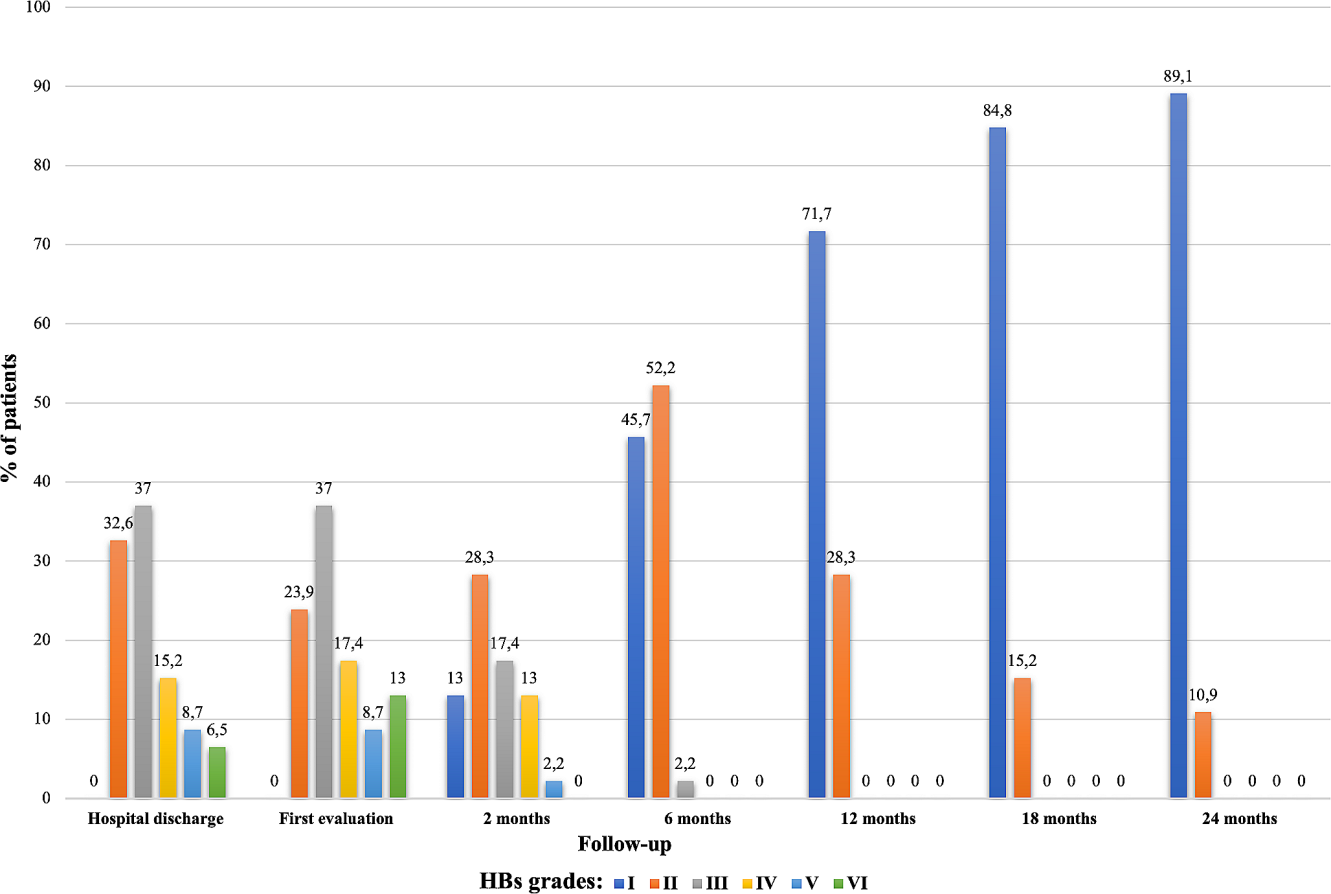
Evolution of facial palsy according to the House-Brackman scale (HBs) during the follow-up

**Fig. 3:**
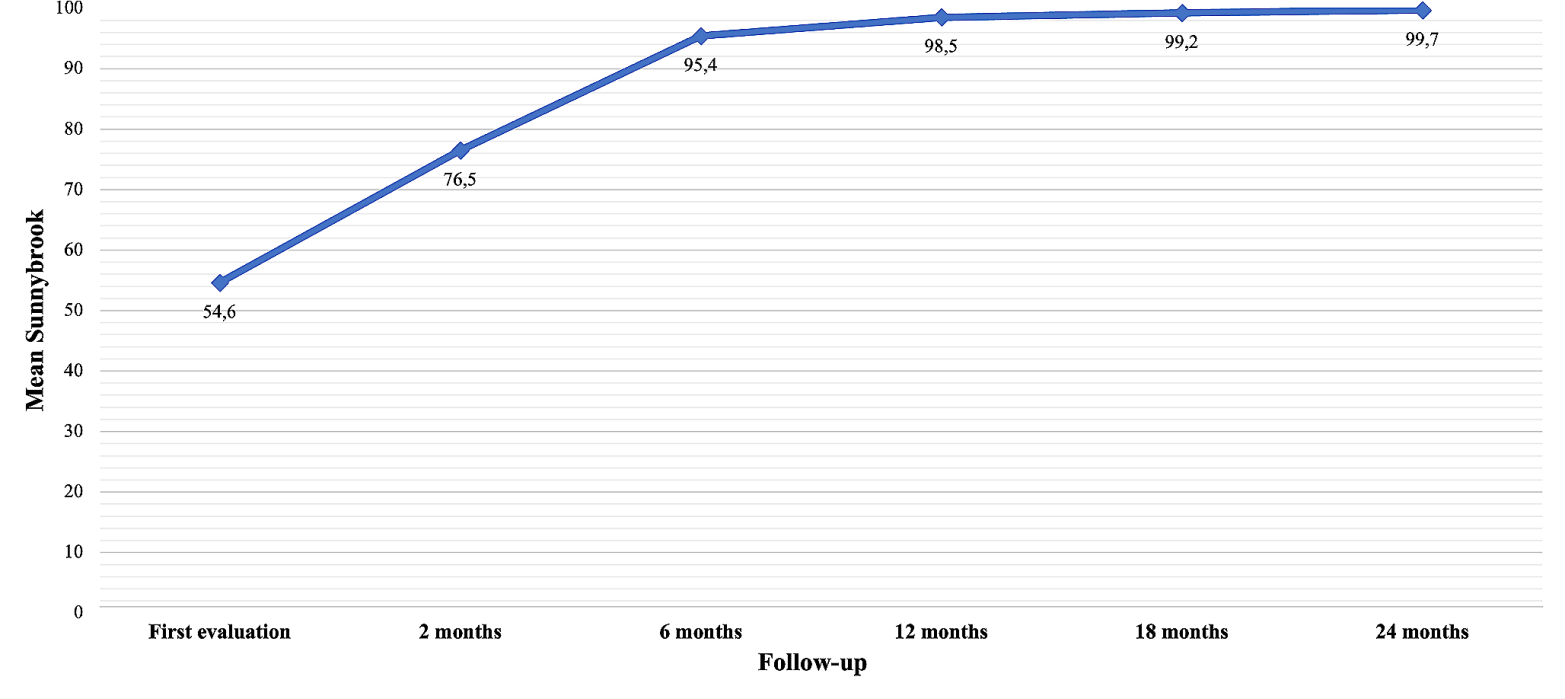
Evolution of facial nerve palsy according to the Sunnybrook scale, during the follow up

Figure 2 reports the evolution of the facial palsy distribution according to HBs from the first evaluation to the end of follow-up, while Fig. 3 shows the trend of the mean value of SBs during follow-up. At the first evaluation at the FNP rehabilitation clinic, the average Sunnybrook rating was 54.6 and 28 patients (60%) had a mild FNP. A worsening of the FNP compared to the immediate post-operative time was detected using the HBs. The topographical distribution of facial paralysis involved cervical-facial branches in 30 patients, temporo-facial branches in 2 patients, and both major nerve trunks in 14 patients.

At 2-month follow-up, the FN function was retrieved for 34 patients, and the mean value of the Sunnybrook scale had increased to 76.5. The reason for the number of patients not evaluated at 2 months after surgery is due to geographical constraints. However, it should be noted that these same patients underwent NMR at home as per logopedic recommendation. Specifically, 45.7% of patients (21/34) had a mild degree of paralysis and 6 of them (6/34, 13%) had already reached complete recovery. Six months after surgery and NMR, Sunnybrook’s mean value was 95.4. Furthermore, 21 patients had fully recovered from facial paralysis, and the highest degree of deficit in the cohort was grade III according to HBs (24 patient grade II and 1 patient grade III). At 12-month follow-up, the full recovery rate of FN function was 72% (33/46) and the worst degree of paralysis was grade II according to HBs (13/46). At 18- and 24-month follow-up, the full recovery rate was 85% and 89% respectively. Two years after surgery, only five cohort patients (10.9%) had persistent grade II paralysis according to HBs. According to the T-test with paired samples (Table 2), the improvement in facial nerve function according to SBs and HBs was statistically significant when comparing between paired follow-up periods up to 18 months (*p* < 0.05). On the contrary, when comparing FN function between 12 and 18, and between 18 and 24 months, no significant difference was detected. When considering the Sunnybrook scale with independent T-test, a statistically significant recovery of patients with moderate-severe paralysis, was observed in the first 2 months of rehabilitation (Table 3). In summary, after an early worsening between discharge and first evaluation at the FNP rehabilitation clinic, a progressive improvement in facial function was noticed, which was greater during the first 6 months of follow-up and reached a plateau after 18 months.

**Table 2:** Paired *T*-test according to paired facial nerve function evaluations during follow-up, using House-Brackman scale and Sunnybrook sale

Paired-sample *t*-test		HBs	SBs
Follow-up		*p*-value	*p*-value
First evaluation	2 months	< 0,001*	< 0,001*
2 months	6 months	< 0,001*	< 0,001*
6 months	12 months	< 0,001*	< 0,001*
12 months	18 months	0,039*	0,012*
18 months	24 months	0,351	0,075

**Table 3:** Independent-samples *t*-test of facial nerve function using the Sunnybrook scale

Independent-samples *t*-test	
Follow-up	*p*-value
First evaluation	< 0,001*
2 months	< 0,001*
6 months	0,254
12 months	0,636
18 months	0,747

### Medical therapy

The rehabilitation process was supported by medical therapy based on oral steroids with decalage (prednisone 1 mg/kg/die) for a mean time of 13 days (7–30 days) and vitamin B complex oral supplements for a mean time of 20 days (15–30 days). This therapy was administered in most of the patients (82,6%) with moderate-severe FNP according to HBs.

We observed a significant difference of recovery after 2 months, in favour of patients not treated with medical therapy, which, however, disappeared after 6 months. The difference between Sunnybrook global value between patients undergoing medical treatment at 2 months at 6 months (and other timepoints) was tested with the Mann-Whitney test. The significance was 0.045 at 2 months and 0.89 at 6 months.

### Univariate and multivariate analysis of other predictors

**Fig. 4:**
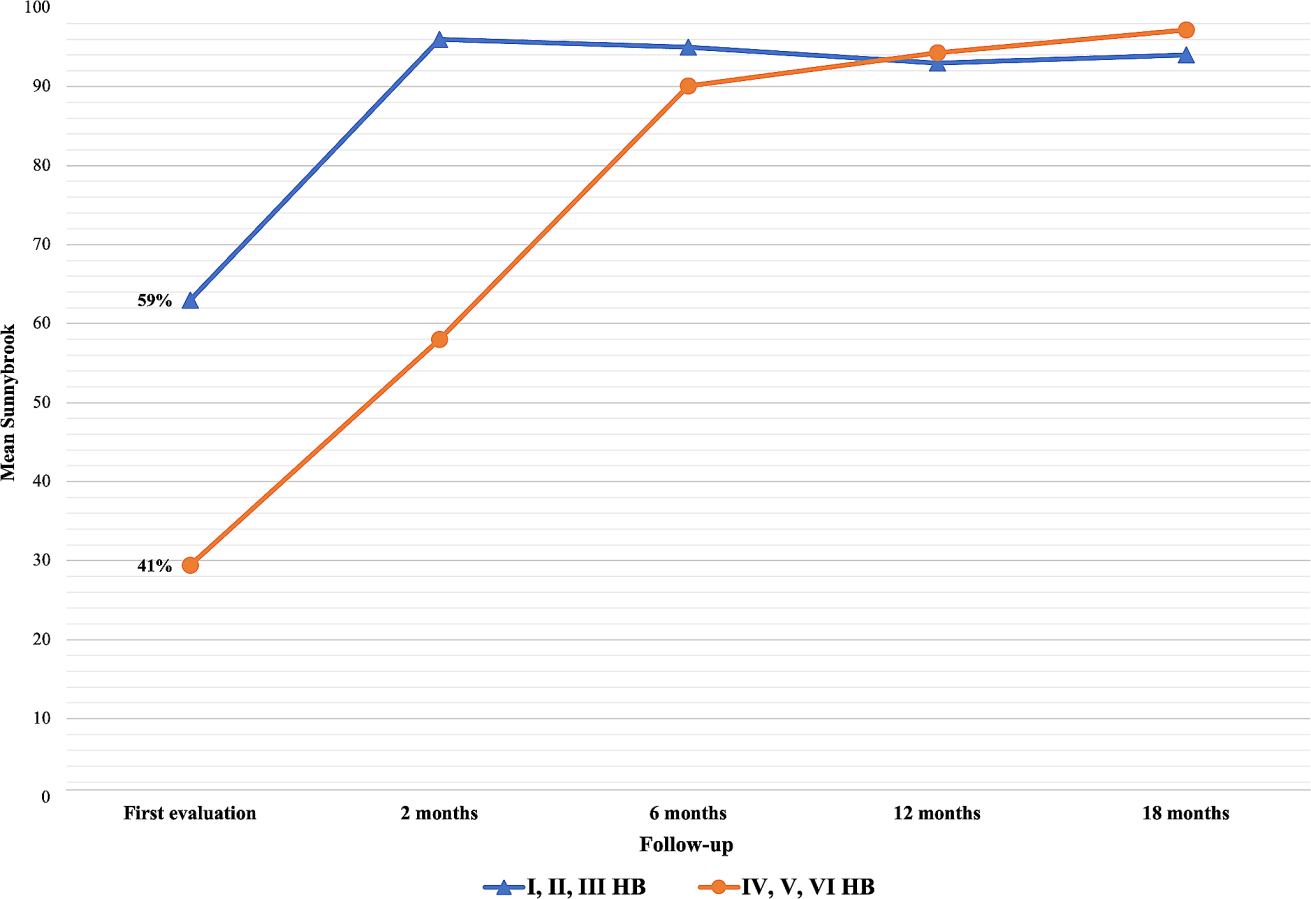
Trends of recovery from facial nerve palsy with neuromuscular retraining, comparing patients affected by mild (equal or less than grade III) to severe (higher than grade III) facial palsy

To identify the possible factors with an impact on facial palsy improvement during the follow-up, the following variables were included in the univariate analysis: severity of the paralysis, age at time of surgery, sex of patient, type of parotidectomy, responsiveness of the facial nerve to NIM at the end of surgery. The results of the FNR in patients with mild vs. severe paralysis according to HBs are reported in Fig. 4, which shows a difference between the average HB values of the patients with mild and severe FP until 2-month follow-up. At 6-month follow-up the two groups showed similar HBs grading distribution. This data was confirmed by the T-test with independent samples analysis on mean values of SBs, for which a steep recovery of patients with severe paralysis was found within the first two months, reaching similar average SBs values between the two groups at 6-month evaluation (Tables 2 and 3). Eventually, the functional recovery of the FN during the different timepoints of the follow-up was not influenced by the age at time of surgery, the sex of patient, the type of parotidectomy, the responsiveness of the FN to NIM at the end of surgery, nor the medical treatment of the paralysis. In addition, the multivariate analysis with linear regression showed no significant impact of the type of surgery, radiotherapy, sex, age, time from surgery to the rehabilitation beginning and at any timepoint from 2 to 24 months. The only variable which resulted a significant predictor at 2 months was a mild (HB *≤* 3) vs. moderate-severe palsy (HB > 3) at T0, even when weighted for patient’s age. The same variable did not prove to be a significant predictor in all other timepoints.

## Discussion

Facial nerve palsy is a major complication of parotid surgery, which can occur even in case of intraoperative preservation of nerve integrity. The lack of voluntary (motor) and involuntary (emotional) facial expression, deficits in stomatognathic functions (phonation, chewing, swallowing, yawn, smile, bite) and eyelid function are among the consequences of FNP. Furthermore, failure to treat FNP may be associated with incomplete or aberrant nerve regeneration, with the development of dysfunctional facial movements, which could further impair patient’s quality of life. To maximize the patient’s functional recovery process, some recovery strategies have been developed, among which NMR. This rehabilitation system was developed in the Netherlands in the 1970s, as a non-surgical therapy for facial movement recovery and the prevention of muscle atrophy, whether used individually or as a complement to surgical treatment [11]. Through the rehabilitation of NMR, based on sensory stimulation, passive and active muscle exercises, and biofeedback, it is possible to progressively promote the reorientation of neural connections and the development of new ways of control of facial muscles, by strengthening existing synapses or synaptogenesis. The therapeutic intervention strategy for acute FNP includes four basic therapeutic phases: (i) initiation; (ii) facilitation; (iii) movement control; (iv) relaxation. This retrospective multicentre study evaluates the evolution of FNP in patients who underwent parotid surgery with intraoperative nerve preservation, rehabilitated with NMR methodology. During the follow-up, a trend of improvement in both HBs and SBs values was observed. First, different grades of paralysis according to HBs scale were recorded (as shown in Fig. 2) while after 6 months, only mild paralysis (grades II and III HBs) were identified, and at the end of the follow-up, complete resolution of the paralysis was obtained in 89,1% of cases. A statistically significant difference in the values recorded between paired follow-up sessions was found during the first year after surgery, for both facial grading systems. In addition, a statistically significant improvement in FNP even in the following interval (12–18 months), while in the last interval considered (18–24 months) no improvement was shown for both facial grading systems. These results allow some interesting considerations regarding the timing and the percentage of recovery to be expected in the rehabilitation from FNP. According to different studies, in most cases of mild FNP, spontaneous improvement in the first year after the onset of the paralysis is possible [12]. Then, spontaneous improvements are limited, and other therapeutic strategies should be offered to the patients [13]. Data regarding the pattern of recovery could enhance preoperative counselling: assessing the impact of facial nerve rehabilitation (FNR) on different degrees of facial nerve paralysis (FNP) could elucidate if such treatment is appropriate even in mild cases and how the frequency of the rehabilitation sessions could be targeted to the patient [14, 15]. According to the literature, this study confirms that even with NMR rehabilitation, maximum improvement is achieved within the first year, but with the application of NMR rehabilitation treatment there is a possibility of improvement even up to 18 months. Beyond 18 months, it is appropriate to evaluate the grading of paralysis achieved by the single patient to define the prosecution of treatment and to consider some surgical approaches (dynamic or static techniques) or chemodenervation. Another important consideration can be done by comparing mild and severe paralysis trends. From the analysis of the trends of mean values according to SB, a substantial difference between the two patient groups existed only until the first follow-up performed at 2 months. This finding suggests how during NMR rehabilitation treatment, patients with moderate-severe paralysis experienced rapid improvement within the first 6 months of rehabilitation, and thereafter maintained a trend comparable to that of patients with mild-to-moderate paralysis, as evidenced by the similar mean SB scores. This finding is meaningful, because one can expect a complete recovery of FP in cases of mild neural injury, while complete recovery is less constant when severe facial palsy occurs after surgery, and some degree of synkinesis could be expected in these patients in the long term. The occurrence of complete or almost complete recovery at 18 and 24 months in the moderate to severe FP group, supports the efficacy of NMR in reducing or preventing synkinesis, as showed by other studies It was found that the portion of the face most affected by paralysis is the one innervated by the cervical-facial branch of the nerve, at the level of the marginalis mandibulae nerve. The preponderance of paralysis in this site is probably due to the anatomical peculiarities of this nerve branch (thin diameter, long course, and lack of anastomotic arches with other branches), according to literature data [13–16]. Among the other characteristics considered, the patient’s gender and the age at time of surgery proved to be variables not significantly correlated with recovery of the facial nerve palsy according to the SB scale. Advanced age is commonly a risk factor for the development of post-operative complications, while young patients, even in post-parotidectomy complications, have better functional reserves and healing abilities than older patients [17]. From this evidence it might seem that NMR exploits patterns of recovery of nerve function that are not strictly dependent on gender and age, even if further studies are required to confirm such results. It could be observed a more significant improvement in young patients, and in this case, a modulation of NMR intensity could be proposed according to age. Analysis of the trend of paralysis during follow-up did not even demonstrate a direct correlation with the type of intervention performed. This suggests that the pattern of improvement could be influenced more by the degree of the paralysis at onset than by the extension of surgery. Finally, all patients included in this study were surgical monitored by using the NIM. Recovery during follow-up was investigated by comparing responsive and nonresponsive patients to NIM at the end of surgery; however, no statistically significant correlations were found between these two groups of patients.

Our study has even some limitations. Firstly, the lack of a control cohort, which would have allowed to compare the results obtained with a group of patients who did not undergo NMR, in order to determine the real added value of this treatment in the selected patients. Thus, a comparison between NMR with other rehabilitation methods may be desirable in future studies. In the present form, this study represents a snapshot of the improvement of FNP in a cohort of strictly selected patients who underwent the same type of rehabilitation, across a 24-month period. Furthermore, the limited number of patients included in the study, as well as the slight heterogeneity related to the different follow-up of each patient, and the lack of a validated objective tool for the assessment of facial nerve palsy, represent other important limitations. In the future, controlled studies including larger cohorts of patients may elucidate how NMR could be tailored to the different patients affected by iatrogenic FNP.

## Conclusion

In our cohort NMR was associated with a complete recovery rate as high as 89,1% and a near-complete recovery in the remaining patients. The negligible rate of synkinesis in the long term, even in patients with severe post-operative FP may support the use of NMR in patients with FP after parotid surgery. The greater rate of functional recovery is achieved within the first 6 months of rehabilitation, but significant improvement is observed until 18 months. Thus, a longer rehabilitation time may be beneficial in patients with incomplete recovery after 12 months. Further controlled studies are warranted to assess the real impact of NMR on these patients.
